# The Variation of Root Exudates from the Hyperaccumulator *Sedum alfredii* under Cadmium Stress: Metabonomics Analysis

**DOI:** 10.1371/journal.pone.0115581

**Published:** 2014-12-29

**Authors:** Qing Luo, Lina Sun, Xiaomin Hu, Ruiren Zhou

**Affiliations:** 1 School of Resources and Civil Engineering, Northeast University, Shenyang, China; 2 Key Laboratory of Regional Environment and Eco-Remediation (Ministry of Education), Shenyang University, Shenyang, China; 3 College of Life Science, Nankai University, Tianjin, China; University of Navarra, Spain

## Abstract

Hydroponic experiments were conducted to investigate the variation of root exudates from the hyperaccumulator *Sedum alfredii* under the stress of cadmium (Cd). *S. alfredii* was cultured for 4 days in the nutrient solution spiked with CdCl_2_ at concentrations of 0, 5, 10, 40, and 400 µM Cd after the pre-culture. The root exudates were collected and analyzed by GC-MS, and 62 compounds were identified. Of these compounds, the orthogonal partial least-squares discrimination analysis (OPLS-DA) showed that there were a distinct difference among the root exudates with different Cd treatments and 20 compounds resulting in this difference were found out. Changing tendencies in the relative content of these 20 compounds under the different Cd treatments were analyzed. These results indicated that trehalose, erythritol, naphthalene, d-pinitol and n-octacosane might be closely related to the Cd stabilization, phosphoric acid, tetradecanoic acid, oxalic acid, threonic acid and glycine could be attributed to the Cd mobilization, and mannitol, oleic acid, 3-hydroxybutanoic acid, fructose, octacosanol and ribitol could copy well with the Cd stress.

## Introduction

Root exudates from a plant are plant metabolites that are released to root surfaces or into the rhizosphere to enhance plant nutrient uptake or copy with environment stresses [1]–[3]. They are generally classified into two types, namely, high molecular weight (HMW) and low molecular weight (LMW) materials. The former includes mucilage (mainly polysaccharides and polyuronic acid) and ectoenzymes; the latter mainly consists of organic acids, sugars, phenols and various amino acids, including non-protein amino acids such as phytosiderophores [4]. Root exudates play an important role in the process of phytoremediation as an emerging green and in-situ remediation technology using plants to absorb, accumulate, stabilize or volatilize contaminants from soil [5]–[7]. Phytoremediation has become a research focus recently because it is a cost-effective and environment friendly technique [6], [8].

Root exudates can react with heavy metal ions and affect metal solubility, mobility, and phytoavailability [9]–[11]. Especially, low molecular weight organic acids (LMWOAs), have strong ability to bind with heavy metals [12]–[15]. Root exudates from *Echinochloa crusgalli*, especially citric acid and oxalic acid, were able to enhance translocation of heavy metals such as Cd, Cu and Pb from roots to shoots [16], [17]. The presence of Cd resulted in reduced phytosiderophore production to enhance transition metal uptake for both barley and wheat plants [18]. Root exudates could also increase solubility of metal ions in soils and consequently increase their accumulation in plants [18]–[21].

However, root exudates released by the Cd- and Zn- hyperaccumulator *Thlaspi caerulescens* do not significantly enhance metal mobilization [22]. Pb could be immobilized in soil when it formed complexes with root exudates, particularly mucilage components [23], [24]. Both plants (Scots pine) and their ectomycorrhizal (ECM) fungi released more LMWOAs such as oxalate that can be contributed to the formation of metal complexes and then immobilized heavy metals under metal stress [25].

*Sedum alfredii* is a Zn/Cd hyperaccumulator native to China, with large biomass, rapid growth, and asexual propagation [26], [27]. Some special substances of root exudates from the hyperaccumulating ecotype of *S. alfredii* could activate Pb and Zn in mined soils, thus increasing their mobilization and bioavailability [28]. Dissolved organic matter (DOM) derived from the rhizosphere of *S. alfredii* could be significantly reduce Zn and Cd sorption and increase their mobility through the formation of soluble DOM-metal complexes [29]. LMWOAs, as important components of root exudates, could enhance heavy metal accumulation in shoots of *S. alfredii* [30], [31]. Citric acid or oxalic acid might be acted as a ligand with Zn to reinforce Zn absorption, transportation and immobilization in roots [32].

At present, some components of root exudates from many plants such as rice and *Flaveria bidentis* can be analyzed by the gas chromatography-mass spectrometry (GC-MS) [33], [34]. However, previous studies were mostly focused upon the roles of root exudates, and changes in the total amount of DOM or DOC, or some specific organic acids and amino acids, detailed components of root exudates from a hyperaccumulator were rarely revealed. The knowledge of root exudates from a hyperaccumulator is advantageous to explain the role of root exudates and to understand mechanisms of mobilizing or immobilizing heavy metals in root exudates.

The hyperaccumulator *S. alfredii* was cultured in Cd stressed nutrition solutions and their root exudates were collected and analyzed by the GC-MS associated with the global analysis in this study. Metabonomics, a high throughput and unbiased comprehensive analysis method, was then used to investigate the variation of root exudates from *S. alfredii* under the stress of Cd.

## Material and Methods

### Chemicals and instruments

Methanol (HPLC grade, Fisher), pyridine (HPLC grade, Sinopharm), methoxamine hydrochloride (Sigma), N-methyl-N-trimethylsilyl trifluoracetamide (MSTFA, Sigma), nitric acid, hydrofluoric acid, and perchloric acid (Sinopharm) were employed in the work. Compositions of the nutrient solution, Na_2_-EDTA and CdCl_2_ were purchased from Sinopharm.

GC-MS (TRACE GC Ultra-PolarisQ, ThermoFisher), the nitrogen purging instrument (MG-2200, TOKYO RIKAKIKAI CO.LTD), and the vacuum freeze drying system (FDU-1100, TOKYO RIKAKIKAI CO.LTD) were used in this study.

### Plant materials and growth conditions

The plant materials of *S. alfredii* were collected from an old Pb/Zn mining area in Quzhou City, Zhejiang Province, China. The sampling site, a public land, is located at 118°, 56' east longitude and 29°, 17' north latitude. It refers to the previous study for more information about this plant [26]. We state that no specific permissions were required for this location. And we confirm that the field studies did not involve in endangered or protected species. This location is a public land, not a private land. We could collect plants in this location without any restrictions. After having collected the plants, the rest experiments were undertaken in our laboratory. The shoot tops of *S. alfredii* were cut and cultured in a greenhouse for 2 months. Healthy and uniform *S. alfredii* seedlings were selected and planted in the basal nutrient solution. The nutrition solution used was the half-strength Hoagland-Arnon solution [35], which comprised of 3 mM KNO_3_, 0.5 mM NH_4_H_2_PO_4_, 2.0 mM Ca(NO_3_)_2_,1.0 mM MgSO_4_·7H_2_O, 4.5 µM MnCl_2_·4H_2_O, 23 µM H_3_BO_3_, 0.4 µM ZnSO_4_·7H_2_O, 0.15 µM CuSO_4_·5H_2_O, 0.05 µM H_2_MoO_4_·H_2_O, and 22 µM EDTA-Fe. The nutrient solution was aerated continuously and renewed every 4 days, with its pH adjusted to 6.0 using 0.1 M NaOH or HCl every day. The plants were grown under greenhouse conditions with natural light, temperature from 10 to 20°C. Until the relatively flourishing roots grow out, also were two weeks of pre-culture, *S. alfredii* plants were selected for 5 Cd treatments: 0 (control), 5, 10, 40, and 400 µM Cd, and Cd was supplied as CdCl_2_. There were 55 pots (1 piece per pot) in total, with 11 replicates for each Cd treatment.

### Collection of root exudates and plant samples

After having grown for 4 days in the nutrient solution spiked with Cd salts without renewal, the plants were transplanted to sterilized pots with 50 mL deionized water per pot to collect root exudates for 6 h. The root exudates from each pot were frozen in liquid nitrogen and freeze-dried for 2 days. The dried residue was resuspended in 100 mL of deionized water and freeze-dried again. The dried residue was redissolved in 10 mL of cold MeOH, then blown to dryness under a gentle nitrogen flow, and reconstituted in 1 mL of n-hexane used for the GC-MS analysis.

After the collection of root exudates, the plant roots in each pot were washed with 100 mL of deionized water and immersed in 20 mM Na_2_-EDTA (disodium ethylenediaminetetraacetate) for 15 min to remove Cd adhering to the root surfaces [36]. The roots and shoots were then harvested separately. The fresh shoots and roots were washed with deionized water, air-dried to remove the water adhering to them, dried at 70°C for 72 h, and then their dry weight was determined. The dried samples were powdered and digested using the HNO_3_-HClO_4_ method and the concentration of Cd was determined by the atomic absorption spectroscopy.

### GC-MS analysis of root exudates

The sample derivation and GC-MS analysis were based on Lisec et al. (2006) [37]. Samples were derivatized by 40 µL of methoxyamine hydrochloride (20 mg mL^−1^ in pyridine, 2 h, 37°C) and 70 µL N-methyl-N-(trimethylsilyl) trifluoroacetamide (MSTFA) (30 min, 37°C). 1 µL of the sample was injected into the GC in the splitless mode. The GC analysis was carried out on a TR-5MS with integrated guard column (30 m, 0.25 µm, 0.25 mm, Thermo Fisher, USA). The injection, interface and ion-source temperatures were adjusted to 230, 250 and 210°C, respectively. The gas flow rate was 1 mL min^−1^, the column temperature was held for 1 min at 70°C, 6 min ramp to 76°C, 50 min ramp to 330°C, 10 min at 330°C. The column end was introduced into an ion trap mass spectrometer. Mass spectra were recorded at 2 scans s^−1^ with a m/z 50–600 scanning range.

### Data analysis

Dry weight data of plants and Cd concentrations were analyzed using one-way ANOVA with the Dun-can's test (*P<0.05*) and SPSS 19.0 for Windows and Excel.

The raw GC-MS chromatogram was automatically analyzed using the automatic mass spectral deconvolution and identification system (AMDIS), and compared with the database of metabolites in plants (Fiehn and GMD). If the similarity was greater than 70%, the compounds could be identified. After the AMDIS output was extracted and processed using the MET-IDEA, 62 compounds were detected in one GC-MS scan. After having normalized the peak area of the identified root exudates, they were imported to a computer using the statistics software SIMCA-P 13.0. The principal component analysis (PCA) and the orthogonal partial least-squares discrimination analysis (OPLS-DA) were used to analyze the variation of root exudates from *S. alfredii* under the Cd stress.

## Results

### Plant growth and Cd accumulation

*S. alfredii* could normally grow when Cd levels ≤40 µM, and there were no visual toxic symptoms. However, some toxic symptoms such as slight necrosis and browning of root tips appeared when these plants were exposed to higher Cd levels, i.e. 400 µM or higher. Root and shoot dry matter yields of the plants increased with an increase in Cd levels (≤40 µM Cd), but a decrease in root and shoot biomass occurred at 400 µM Cd or higher (Table 1). There are no statistically significant differences (*P<0.05*) in dry weight of roots and shoots between the treatments of 400 µM Cd versus 0, 5, 10, or 40 µM Cd.

**Table 1:** Changes in dry weights, Cd concentrations and Cd accumulation in various tissues of *S. alfredii* with exposure to supplying Cd levels for 4 days.

	Dry weight (n = 11)		Cd concentration (n = 11)			Cd accumulation (n = 11)	
Cd level (µM)	Shoot	Root	Shoot	Root	Transfer coefficient	Shoot	Root
	mg plant^−1^		mg kg^−1^ DW			µg plant^−1^	
0	165.9±12.8 b	13.3±0.27 a	131.5±4.25 e	45.8±1.87 e	2.87	21.5±2.04 e	0.59±0.02 d
5	182.0±19.5 ab	14.2±0.97 a	778.8±17.7 d	86.9±4.24 d	8.96	120.6±8.01 d	1.16±0.09 d
10	197.2±19.3 ab	14.8±1.34 a	1333.1±32.7 c	150.2±5.05 c	8.87	261.4±24.2 c	2.22±0.21 c
40	239.4±22.8 a	17.2±1.60 a	2015.1±20.7 b	190.6±7.67 b	10.6	483.2±47.5 b	3.23±0.27 b
400	218.0±13.2 ab	15.1±0.33 a	3997.3±54.1 a	997.8±20.7 a	4.10	869.6±52.3 a	15.0±0.40 a

Different letters indicated the difference of the same column at *P<0.05*;

Cd accumulation  = Cd concentration × Dry weight

The Cd concentrations in roots and shoots of *S. alfredii* increased rapidly with an increase in external Cd supply levels, when having peaked at ≤400 µM Cd. The maximum Cd concentrations in roots and shoots reached 997.8 and 3997.3 mg kg^−1^(DW), respectively (Table 1). Similarly, Cd uptake by shoots and roots linearly increased with an increase in Cd supply levels, when having peaked at ≤400 µM Cd. The maximum amount of Cd taken up by shoots was as high as 869.6 µg plant^−1^. The maximum amount of Cd taken up by roots was 15.0 µg plant^−1^, only one sixtieth of that by shoots (Table 1). There were significant differences (*P<0.05*) in Cd concentrations and accumulation in roots and shoots between the treatments of 400 versus 0, 5, 10, or 40 µM Cd. The transferring coefficient (the ratio of Cd concentrations in shoots and roots) of *S. alfredii* ranged from 2.87 to 10.6 (Table 1).

These results confirmed that *S. alfredii* had higher Cd requirement and an amazing ability to tolerate Cd and its transport to shoots, which might be a result of strong selection pressure under high soil heavy metal concentrations in mined areas.

### Composition of root exudates under different Cd stresses

**Figure 1:**
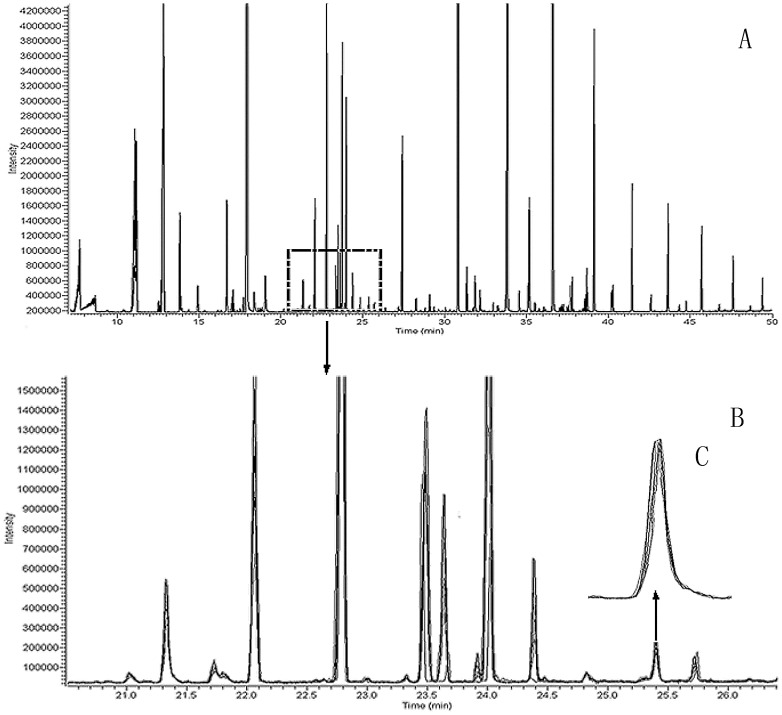
Repeatability of the data.

Through sampled and injected the same sample for seven times, the chromatogram was no significant drift and the relative standard deviation (RSD) is less than 10% (Fig. 1). Fig. 1A is the overlapping chromatograms, Fig. 1B is the enlarged one of the selected portion, and Fig. 1C is the enlarged one of the selected peak. These consistent data ensured the reliability and accuracy of the subsequent analysis results.

The root exudates from *S. alfredii* at different Cd exposed concentrations were analyzed using the method of 2.4 and 2.5 and the results are depicted in Table 2. The 62 compounds including organic acids, amino acids and sugar were identified in one GC-MS scan by the mass spectrum and the retention time. Some of these compounds are the same compound with the different retention time.

**Table 2:** The identified compounds of root exudates from *S. alfredii*.

ID	RT	Identity	Cd exposed concentration(µM)				
			0	5	10	40	400
1	8.44	Oxalic acid (2TMS)	1.00±0.16	1.09±0.11	0.00±0.00	0.00±0.00	0.00±0.00
2	11.09	Lactic acid (2TMS)	1.00±0.17	0.96±0.13	0.81±0.14	0.84±0.14	0.98±0.07
3	11.35	Hexanoic acid (1TMS)	1.00±0.20	1.33±0.21	1.37±0.19	1.17±0.16	0.98±0.17
4	11.59	2-Hydroxyacetic acid (2TMS)	1.00±0.13	0.87±0.08	0.69±0.05	0.82±0.10	0.84±0.17
5	13.57	Oxalic acid (2TMS)	1.00±0.23	1.90±0.86	0.53±0.18	0.00±0.00	0.00±0.00
6	14.01	p-Cymene	1.00±0.40	0.00±0.00	0.00±0.00	0.00±0.00	0.00±0.00
7	14.04	Phenylmethanol (1TMS)	0.00±0.00	1.21±0.29	1.00±0.18	0.00±0.00	0.00±0.00
8	14.38	3-Hydroxybutanoic acid (2TMS)	1.00±0.38	0.00±0.00	0.00±0.00	0.00±0.00	0.00±0.00
9	14.48	Heptanoic acid(1TMS)	1.00±0.16	1.04±0.18	0.91±0.09	0.86±0.07	0.35±0.07
10	15.07	Naphthalene	1.00±0.35	0.31±0.08	0.32±0.05	0.00±0.00	0.13±0.04
11	16.1	L-Valine (2TMS)	1.00±0.22	0.80±0.17	0.54±0.06	0.54±0.03	1.04±0.17
12	16.86	Urea (2TMS)	1.00±0.19	1.12±0.17	3.68±0.76	0.78±0.08	2.03±0.38
13	16.87	Benzoic acid (1TMS)	0.00±0.00	0.00±0.00	0.00±0.00	1.00±0.06	0.00±0.00
14	16.92	Diethyleneglycol (2TMS)	1.00±0.34	1.46±1.07	0.00±0.00	0.33±0.03	0.00±0.00
15	17.36	Octanoic acid (1TMS)	1.00±0.14	1.19±0.22	0.93±0.13	1.20±0.10	1.22±0.14
16	17.8	L-Leucine (2TMS)	0.00±0.00	1.00±0.71	0.00±0.00	0.00±0.00	0.00±0.00
17	17.91	Phosphoric acid (3TMS)	1.00±0.13	0.62±0.11	0.45±0.11	0.84±0.22	3.08±0.83
18	17.96	Glycerol (3TMS)	1.00±0.28	0.45±0.08	0.33±0.05	0.33±0.06	0.15±0.02
19	18.72	Glycine (3TMS)	1.00±0.21	1.80±0.26	0.96±0.22	0.83±0.17	1.16±0.18
20	18.89	Succinic acid (2TMS)	1.00±0.31	0.83±0.09	0.77±0.11	1.20±0.22	1.11±0.22
21	19.54	Glyceric acid (3TMS)	1.00±0.37	0.76±0.13	0.63±0.13	1.25±0.57	0.70±0.11
22	19.79	Fumaric acid (2TMS)	0.00±0.00	1.00±0.12	0.00±0.00	0.00±0.00	0.00±0.00
23	20.06	Nonanoic acid (1TMS)	1.00±0.09	0.88±0.11	0.76±0.11	1.10±0.09	1.01±0.05
24	20.16	L-Alanine (3TMS)	1.00±0.29	1.45±0.22	0.88±0.26	0.56±0.13	1.24±0.41
25	20.37	L-Serine (3TMS)	0.00±0.00	1.00±0.23	0.43±0.12	0.37±0.07	0.66±0.11
26	21.54	Putrescine (4TMS)	0.00±0.00	1.00±0.12	1.19±0.07	1.03±0.08	1.13±0.08
27	21.8	Putrescine (4TMS)	0.00±0.00	1.00±0.06	1.10±0.10	1.09±0.06	1.00±0.07
28	22.08	Putrescine (4TMS)	0.00±0.00	1.00±0.04	1.08±0.06	0.00±0.00	1.12±0.10
29	22.58	Decanoic acid (1TMS)	0.00±0.00	0.00±0.00	0.00±0.00	1.00±0.14	0.90±0.05
30	22.79	Putrescine (4TMS)	1.00±0.09	1.11±0.08	1.17±0.09	1.15±0.07	1.17±0.08
31	24.27	Erythritol (4TMS)	1.00±0.14	0.31±0.03	0.14±0.03	0.00±0.00	0.15±0.01
32	24.81	2,4,6-Tri-tert.-butylbenzenethiol	1.00±0.09	0.58±0.09	0.47±0.06	0.49±0.06	0.54±0.04
33	25.16	Threonic acid (4TMS)	0.00±0.00	1.00±0.11	1.43±0.22	0.00±0.00	0.00±0.00
34	25.26	Dodecanol(1TMS)	1.00±0.20	1.12±0.20	0.83±0.14	1.17±0.18	0.63±0.10
35	25.61	Threonic acid (4TMS)	1.00±0.36	4.15±1.49	1.59±0.55	1.08±0.21	0.00±0.00
36	27.2	Dodecanoic acid (1TMS)	1.00±0.09	1.32±0.19	1.23±0.23	2.13±0.26	2.18±0.15
37	28.34	Xylose (4TMS 1MEOX)	0.00±0.00	0.00±0.00	0.00±0.00	0.00±0.00	1.00±0.44
38	29.24	Ribitol (5TMS)	1.00±0.14	0.34±0.04	0.00±0.00	0.11±0.02	0.00±0.00
39	29.81	Diethyleneglycol (2TMS)	1.00±0.24	0.95±0.32	0.00±0.00	0.00±0.00	0.00±0.00
40	30.35	Terephthalic acid (2TMS)	0.00±0.00	0.00±0.00	0.00±0.00	0.00±0.00	1.00±0.19
41	30.43	Azelaic acid(2TMS)	1.00±0.14	1.05±0.21	0.00±0.00	0.00±0.00	0.00±0.00
42	31.39	Tetradecanoic acid (1TMS)	1.00±0.23	1.07±0.25	0.93±0.15	1.23±0.28	4.38±0.26
43	32.24	D-Pinitol (5TMS)	1.00±0.11	0.63±0.24	0.55±0.15	0.00±0.00	0.27±0.06
44	32.48	Fructose (5TMS 1MEOX)	1.00±0.17	0.63±0.16	0.31±0.06	0.00±0.00	0.00±0.00
45	32.69	Fructose {BP} (5TMS 1MEOX)	1.00±0.21	0.00±0.00	0.00±0.00	0.00±0.00	0.00±0.00
46	32.99	Glucose (5TMS 1MEOX)	1.00±0.12	0.74±0.13	0.57±0.08	1.05±0.58	0.76±0.12
47	33.59	Mannitol (6TMS)	1.00±0.18	0.30±0.04	0.10±0.03	0.06±0.03	0.09±0.02
48	34.78	9-Hexadecenoic acid(1TMS)	1.00±0.35	0.00±0.00	0.00±0.00	0.00±0.00	0.00±0.00
49	35.26	Hexadecanoic acid (1TMS)	1.00±0.46	0.57±0.06	0.40±0.05	0.44±0.04	0.54±0.06
50	37.2	Octadecanol (1TMS)	1.00±0.14	0.70±0.23	0.82±0.14	0.76±0.20	0.52±0.10
51	37.93	n-Octacosane	1.00±0.09	0.00±0.00	0.00±0.00	0.00±0.00	0.67±0.08
52	38.31	Oleic acid (1TMS)	1.00±0.17	0.86±0.13	0.92±0.18	0.58±0.08	0.00±0.00
53	38.4	9-Octadecenoic acid(1TMS)	0.00±0.00	0.00±0.00	1.00±0.23	0.00±0.00	0.00±0.00
54	38.76	Octadecanoic acid (1TMS)	1.00±0.40	0.56±0.13	0.45±0.06	0.57±0.04	0.77±0.07
55	39.63	n-Docosane	1.00±0.09	0.78±0.13	0.65±0.12	0.57±0.09	0.56±0.09
56	41.27	n-Octacosane	1.00±0.12	0.87±0.14	0.67±0.11	0.59±0.10	0.70±0.09
57	44.4	1-Monohexadecanoylglycerol(2TMS)	1.00±0.19	0.91±0.26	0.59±0.13	1.35±0.52	0.41±0.05
58	47.18	1-Monooctadecanoylglycerol(2TMS)	1.00±0.25	0.73±0.46	0.00±0.00	1.16±0.49	0.00±0.00
59	47.27	Trehalose (8TMS)	1.00±0.15	0.33±0.02	0.22±0.07	0.07±0.02	0.24±0.04
60	51.8	Octacosanol (1TMS)	1.00±0.25	0.00±0.00	0.00±0.00	0.00±0.00	0.00±0.00
61	52.22	Cholesterol (1TMS)	0.00±0.00	1.00±0.31	0.00±0.00	0.00±0.00	0.49±0.07
62	54.62	beta-Sitosterol (1TMS)	0.00±0.00	0.00±0.00	0.00±0.00	0.00±0.00	1.00±0.37

Values were normalized according to each compound based on the first appeared by the following. At first, the peak area values were divided by the average of the compound which first appeared. Then, the means and standard errors (SE) of each compound were calculated according to the treatments. This normalization allowed the principal component analysis and the orthogonal partial least-squares discrimination analysis to extract the exact effect of the increase and decrease of the targeted compounds, regardless of their absolute amounts. MEOX, methoxylation; TMS, trimethylsilylation.

By analyzing the relative peak area of the 62 identified compounds, it showed that the compositions of the root exudates from *S. alfredii* were significantly different at different Cd exposed concentrations.

**Figure 2:**
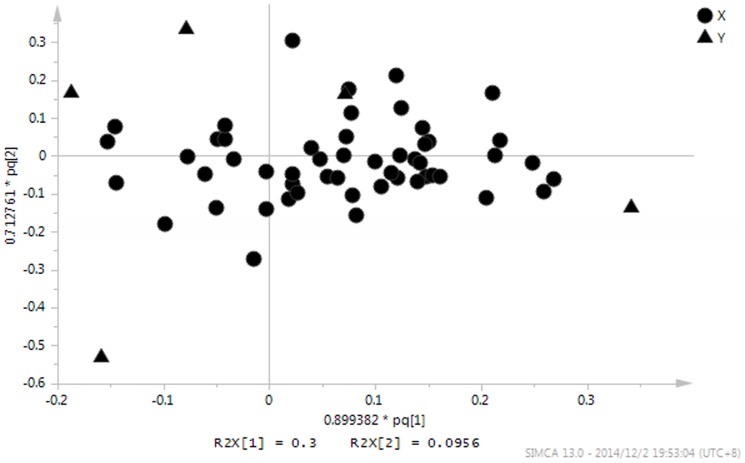
Loadings plot of identified root exudates from *S. alfredii* for the OPLS-DA model.

**Figure 3:**
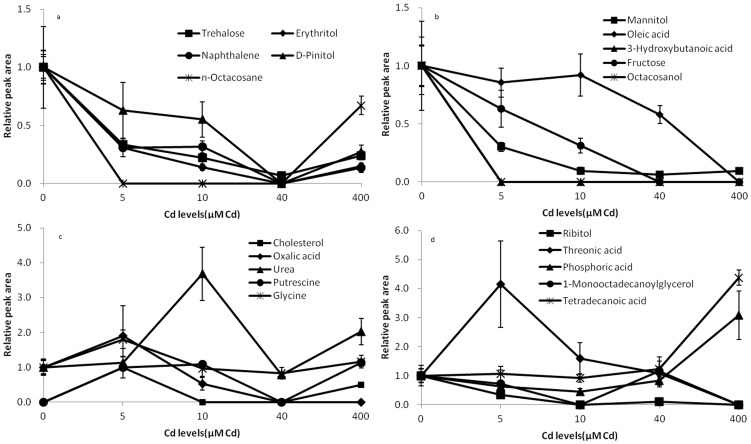
The variation of identified root exudates that caused the separation among the different Cd exposed concentrations.

To find out the compounds of the identified root exudates which resulting in the separation among the different Cd exposed concentrations, the loadings plot of the related OPLS-DA model was conducted (Fig. 2). Combined with the loadings plot, the variable importance factor (VIP) values of the OPLS-DA and analysis of variance (ANOVA), 20 compounds resulting in the separation among the different Cd concentrations were found (Fig. 3). In Fig. 3a, trehalose, erythritol, naphthalene, d-pinitol and n-octacosane secretions decreased with an increase in the concentration of Cd treatments. However, at the concentration of 400 µM, these compounds secretions increased. Mannitol, oleic acid, 3-hydroxybutanoic acid, fructose and octacosanol secretions decreased with an increase in the concentration of Cd treatments (Fig. 3b).

In Fig. 3c, cholesterol, urea, putrescine and glycine secretions increased with an increase in the concentration of Cd treatments at low Cd levels. With further increase of Cd treatments, these compounds secretions decreased. But at 400 µM Cd, these compounds secretions increased. Oxalic acid and threonic acid secretions increased at 5 µM Cd, but decreased with a further increase in the concentration of Cd treatments.

Both ribitol and 1-monooctadecanoylglycerol secretions decreased with an increase in the concentration of Cd treatments at low Cd levels (Fig. 3d). With further increase of Cd treatments, these two compounds secretions increased. But at 400 µM Cd, these two compounds secretions increased. Phosphoric acid secretions decreased with an increase in Cd concentrations at low Cd exposed concentrations, but increased with a further increase in the concentrations of Cd treatments. Tetradecanoic acid secretions increased with an increase in the concentration of Cd treatments.

### Principal component analysis of identified root exudates

The identified root exudates were analyzed using the unsupervised principal component analysis method. A relatively stable model has been obtained through PC1, PC2 and PC3. The explained variations of PC1, PC2 and PC3 were 30.9%, 10.3% and 7.9%, respectively. And the accumulation explained the variation of the model is 49.1%.

**Figure 4:**
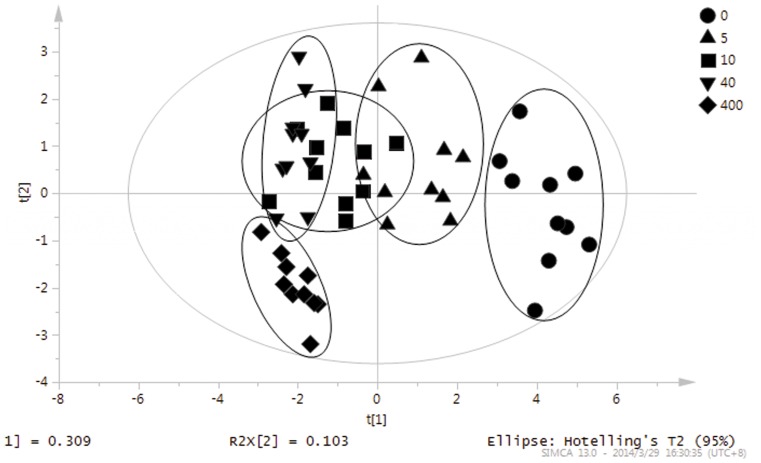
Sample scores for the first (PC1) and second (PC2) principal components from the principal component analysis for identified root exudates from *S. alfredii*.

The results of the PCA on the identified root exudates were described in Fig. 4. The representative points of the samples were mapped in the space spanned by the first two principal components PC1 versus PC2. This scores plot is illustrated a reasonable clustering appearing according to different Cd exposed concentrations. PCA unravelled the existence of differences in the composition of root exudates (30.9% of variance was captured by the first PC) from *S. alfredii* at different Cd exposed concentrations.

### Orthogonal partial least-squares discrimination analysis of identified root exudates

**Figure 5:**
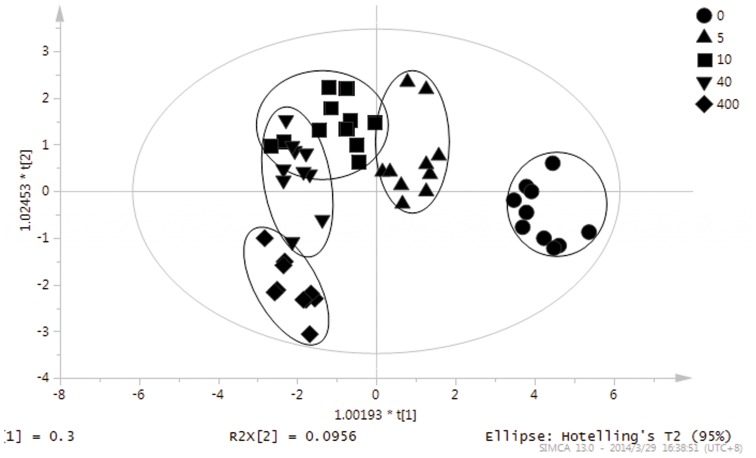
Sample scores of the orthogonal partial least-squares discrimination analysis for identified root exudates from *S. alfredii*.

The supervised clustering method OPLS-DA was carried out to enhance the separation obtained from the PCA model for the identified root exudates. A better separation was attained after OPLS-DA for the identified root exudates under different Cd stresses. The scores plot of the first and the second latent variable was shown in Fig. 5. The accumulation explained variations and predictive variations of the model were 81.4% and 69.2%, respectively.

## Discussion

Greater tolerances of a plant to high concentrations of generally toxic metals, as well as hyperaccumulation of metals in aerial parts of a plant are typical characteristics of a hyperaccumulator [38]–[41]. These characteristics of *S. alfredii* were confirmed in this study, and the results presented are highly consistent with the results from the previous study [26]–[30]. Successful accumulation of these metals in a hyperaccumulator probably requires its root exudates that can mobilize metals and promote their uptake by roots [42], [43]. Therefore, an investigation on the components of root exudates was performed in *S. alfredii* and contrasted with that in different Cd exposed concentrations in order to reveal possible mechanisms of a Cd-hyperaccumulator. Mainly two techniques, the GC-MS associated with the global analysis and the OPLS-DA, were employed to determine components of root exudates and their variations at different Cd exposed concentrations. The obtained results using the GC-MS associated with the global analysis of the components of root exudates from *S. alfredii* indicated that organic acids, amino acids and sugar were the main components of root exudates, which agreed with the previous detection [44]. However, alcohol compounds such as glycerol, erythritol, dodecanol and mannitol were detected in the current study, which indicated that the exudation of alcohol compounds might be an important influencing factor in tolerance and accumulation of a hyperaccumulator [6], [7], [13].

The current evidence suggested that the quantity and composition of root exudates could be influenced by many factors [45] including soil structure [46], presence of microorganisms [47], plant species [48] as well as their developmental stage [43], nutritional status [12] and environmental stresses [3], [6]. In this study, through the PCA and OPLS-DA of the identified root exudates, it detected that the quantity or composition of root exudates released from *S. alfredii* at different Cd exposed concentrations are obviously different. These results proved that hyperaccumulators can change the secretions of root exudates in order to tolerate or accumulate heavy metals further.

It is important to understand which compounds in root exudates play the main role in tolerate or accumulate heavy metals. Liu et al. (2008) [30] added citric and oxalic acids to soil and observed that the ability of *S. alfredii* tolerating and accumulating heavy metals increased. Li et al. (2011) [29] fractionated DOM derived from the rhizosphere of the hyperaccumulator and non-hyperaccumulating ecotype into hydrophilic acid, hydrophilic base, hydrophilic neutral, hydrophobic acid, hydrophobic base and hydrophobic neutral, detected that the hydrophilic fractions (51%) in DOM from the rhizosphere of the hyperaccumulator were much greater than the non-hyperaccumulating ecotype (35%) and suggested that this fractions could significantly reduce metal sorption and increase its mobility. Jiang et al. (2013) [31] detected methane-sulfonic acid, hexane di-acid, citric acid, formic acid, acetic acid and succinic acid in root exudates and found that *S. alfredii* could exuded more methane-sulfonic acid and hexane di-acid to mobilize more Zn than maize. In this study, we found 20 compounds caused the separation among the different Cd exposed concentrations using the loadings plot and the VIP values of OPLS-DA and ANOVA, and suggested that these compounds derived from *S. alfredii* might play a main role in tolerating or accumulating heavy metals.

These compounds include 6 organic acids (oxalic acid, threonic acid, phosphoric acid, oleic acid, tetradecanoic acid and 3-hydroxybutanoic acid), 1 amino acid (glycine), 2 sugars (trehalose and fructose), 6 alcohol compounds (cholesterol, ribitol, erythritol, mannitol, d-pinitol and octacosanol) and 5 other compounds (urea, naphthalene, 1-monooctadecanoylglycerol, putrescine and n-octacosane). Their relative contents showed different changes with an increase of Cd levels.

Compared with the control (0 µM Cd), the secretions of trehalose, erythritol, naphthalene, d-pinitol and n-octacosane reduced in the low Cd exposed levels. However, the secretions of these compounds increased with an increase in the Cd exposed level. These results indicated that these compounds might play an important role in stabilizing Cd [49]. At the low Cd exposed levels, through the reduction of the secretions of these compounds, the amount of available Cd increased more. In other words, *S. alfredii* could absorb more Cd. But in the high Cd exposed levels, through the increase of the secretions of these compounds, the amount of available Cd became less, because *S. alfredii* could avoid being poisoned.

Under the Cd stress, the secretions of mannitol, oleic acid, 3-hydroxybutanoic acid, fructose, octacosanol and ribitol reduced vs. the control, and reduced with an increase in the Cd exposed level. These results indicated that *S. alfredii* could release these compounds to copy with the Cd stress. Under the Cd stress, the secretions of phosphoric acid and tetradecanoic acid increased vs. the control, and increased with an increase in the Cd exposed level. These results indicated that these two compounds may be attributed to mobilization of Cd. Through increasing the secretions of these two compounds, the amount of available Cd became more. In other words, *S. alfredii* could absorb and accumulate more Cd.

The secretions of oxalic acid, threonic acid and glycine increased at first and then descended with an increase in the Cd exposed level. This result indicated that *S. alfredii* exposed to the low Cd levels could release more these compounds to mobilize Cd, thus adding the amount of available Cd, and increasing the adsorption and accumulation of Cd. But with the further increase of the Cd exposed levels, their secretions decreased in order to avoid the poisoning of *S. alfredii*. Due to the changes of the secretions of trehalose, urea, 1-monooctadecanoylglycerol and putrescine were more complex with an increase in the Cd exposed levels. We do not sure whether these compounds play an important role in accumulating Cd.

Due to the limitation of the experiment method, the sensitivity of GC-MS and the library of MS, only 62 compounds were identified in this study. However, the components of plant root exudates are multitudinous and complex [44], [50], [51]. In order to thoroughly understand the role and mechanisms of root exudates from hyperaccumulators that can tolerate and accumulate heavy metals, more excellent methods such as UPLC-Q-TOF or NMR will be needed to detect the components of root exudates in the future. And further study on the role of root exudates which can cause the separation among different environmental stresses should be conducted.
